# Effects of meteorological factors on outpatient visits for chronic rhinosinusitis in Wuhan, China (2018–2019): a time-series analysis

**DOI:** 10.3389/fpubh.2025.1621856

**Published:** 2025-06-27

**Authors:** Xiaoli Guan, Wen Xiang, Ao Huang, Min Zhou, Ming Zeng

**Affiliations:** ^1^Department of Nursing, Tongji Hospital, Tongji Medical College, Huazhong University of Science and Technology, Wuhan, China; ^2^Department of Otolaryngology-Head and Neck Surgery, Tongji Hospital, Tongji Medical College, Huazhong University, Wuhan, China

**Keywords:** chronic rhinitis, meteorological factors, time series analysis, China, outpatient visits

## Abstract

**Background:**

Studies examining associations between meteorological factors and outpatient visits for chronic rhinosinusitis (CRS) are limited. This study aimed to investigate the effects of daily mean temperature, relative humidity (RH) and precipitation on outpatient visits for CRS.

**Methods:**

Electronic records of CRS outpatient visits were collected from Tongji hospital in Wuhan, China from January 1, 2018 to December 31, 2019. Daily meteorological data were obtained from the Wuhan Meteorological Bureau during the same period. A generalized additive negative binomial regression model combined with a distributed lag non-linear model (DLNM) was employed to analyze the lag-exposure-response relationship between meteorological factors and the number of CRS outpatient visits. Stratified analyses were conducted to identify potential effect modifications by age and season.

**Results:**

A total of 14,259 CRS outpatient visits were recorded. Relative humidity and precipitation showed no significant association with daily CRS visits, whereas low temperatures significantly elevated CRS outpatient visits. Specifically, extreme temperature (−1.8°C, 1st percentile) was found to be associated with 1.946 (95% CI 1.273–2.973) times the risk of outpatient visits due to CRS, compared to the reference value of 32.9°C. Furthermore, the number of outpatient visits for childhood and younger individuals with CRS showed a negative correlation with temperatures, whereas middle-aged individuals and older adult individuals showed no such correlation.

**Conclusion:**

This study suggests that meteorological phenomena may have detrimental effects on health, thereby contributing to a better understanding of the environmental risk factors associated with this disease.

## 1 Introduction

Chronic rhinosinusitis (CRS) is one of the most prevalent chronic inflammatory diseases, characterized by a sustained inflammatory response in the paranasal sinuses and nasal cavity lasting at least 12 consecutive weeks (1, 2). Common symptoms include facial pain or pressure, nasal blockage, obstruction or congestion, nasal discharge, and reduction or loss of smell (3). CRS has been found to affect approximately 12% of individuals in the United States, and 11% in Europe, while a prevalence of 8% was reported in China (4). CRS poses a significant global health issue, resulting in diminished quality of life, a substantial symptom burden, and heavy economic costs.

Considering the adverse effects of chronic rhinosinusitis (CRS), recent studies have increasingly focused on the various factors influencing this condition, including genetics, occupational exposure, comorbid diseases, and environmental factors. Several studies have estimated that air pollution is a major risk factor contributing to an increased incidence of CRS (5–7). Key components of air pollution, such as ambient particulate matter (PM), particularlyPM2.5 (6) and PM10 (8), as well as gaseous pollutants including nitrogen dioxide (9), have been shown to pose significant health risks related to CRS. Recent evidence also suggests a combined effect of genetic factors and air pollution on the development of CRS (5). Concurrently, there is growing evidence that workers exposed to gases, fumes, dust, and smoke are at a higher risk of developing CRS (10). Recently, the significance of meteorological factors, such as temperature and relative humidity (RH), has gained recognition, due to their substantial role in the prevalence and severity of respiratory disease (10–14). Additionally, a survey examining the association between high daily mean temperatures and chronic rhinosinusitis in the United States found that exposure to extreme heat was linked to a higher likelihood of exacerbating CRS symptoms (OR 1.11, 95% CI 1.03–1.19) (13). However, a cross-sectional study conducted in Korea from 2002 to 2015 reported several contradictory findings, indicating no positive correlation between the rates of CRS patients and mean, highest, or lowest temperatures, temperature range, or rainfall (11). The discrepancies in the findings can be attributed to several factors, including geographical and climatic differences, variations in exposure assessment methods, and differences in study design. Despite these inconclusive findings, the impact of meteorological factors on the incidence and progression of CRS, especially within the Chinese population, remains unstudied. Considering China’s large population and climatic diversity, there is an urgent need to address the significant research gap regarding the influence of meteorological factors on CRS. Therefore, the aim of this study is to investigate the relationship between meteorological factors (daily mean temperature, relative humidity, and precipitation) and the number of CRS outpatient visits in China.

## 2 Methods

### 2.1 Data sources

We collected data on outpatient visits for CRS during January 1, 2018, to December 31, 2019, using the electronic medical records of the Department of Otolaryngology-Head and Neck Surgery at Wuhan Tongji Hospital. Daily meteorological data, encompassing daily mean temperature (°C), precipitation (mm), and relative humidity (%), were sourced from the National Meteorological Information Center of China^1^ for the same period. Diagnoses of CRS during outpatient visits were performed by qualified otolaryngologists at Tongji Hospital. We reviewed medical records to identify and include patients diagnosed with CRS, adhering to the CRS diagnostic criteria outlined in the European Position Paper on Rhinosinusitis and Nasal polyps (2020) definition (15). Participants were restricted to residents of Wuhan, excluding individuals residing outside the city. For each patient, we collected demographic and clinical characteristics. The data used in this study were anonymized prior to analysis, therefore individual consent was not required. This processing method has been approved by the Ethics Committee of Tongji Hospital of Tongji Medical College, Huazhong University of Science and Technology (TJ-C20190311) and was conducted in accordance with the Declaration of Helsinki.

### 2.2 Statistical analysis

A descriptive analysis was conducted on the demographic data of CRS, including age and type of season, as well as meteorological factors such as daily mean temperature, relative humidity, and precipitation. A time series diagram was plotted to illustrate the trends of these variables over time. Spearman correlation analysis was employed to assess the relationships between meteorological factors and the number of daily outpatient visits for CRS. To evaluate the exposure-response relationships between meteorological factors and outpatient visits for CRS, a generalized additive model (GAM) following a negative binomial distribution, along with a distributed lag non-linear model (DLNM), was utilized. This model accounted for several confounding factors, including meteorological variables, day of the week (DOW), and holidays. The model is defined as follows:

$\begin{array}{l} \log[(Y_{t})]=\beta_{0}+\sum \mathrm{cb}[(\text{meteoro}\log\text{ical}\mathrm{var}\text{iables},df_{1}),(\mathrm{lag},df_{2})]\\ +s(\mathrm{doy},df_{3})+s(t,df_{4})+\text{factor}(\mathrm{dow}) \end{array}$

Where E(Y_t_) represents the adjusted daily outpatient visits on day t; t is the day of the study period from 2018 to 2019 (1,2, … 730); $\beta_{0}$ is the intercept; ^cb^() denotes the cross-basis function in the distributed lag models, which describes the exposure-response and lag-response associations with their corresponding degrees of freedom; s () indicates a thin plate penalized spline function; and DOY represents the day of the year (1, 2, …, 365). Meteorological variables, including daily mean temperature, relative humidity, and precipitation were controlled in the models to account for potential confounding effects. In addition to the year-round analysis, a stratified analysis was performed by age groups (<18 years, 18–45 years, 45–65 years, and >65 years) and by season, specifically the warm season from April through October and the cold season from November through March (16).

The current analysis was conducted on a daily scale, with the maximum period for delayed effects determined to be 30 days (16–18). Different degrees of freedom were applied to all cross-basis and spline functions to assess the robustness of the models. The regression coefficient (R^2^) and Akaike’s Information Criterion (AIC) were utilized for model selection. All associations were reported as the relative risk (RR) of a specified percentile value of a particular meteorological variable, accompanied by the corresponding 95% confidence interval (CI), in comparison to a pre-defined reference value. For instance, the RR for temperature was calculated as the ratio of the likelihood of daily visits when an individual was exposed to a specific temperature value, relative to the probability of daily visits when that individual was exposed to the reference temperature value. All statistical analyses were performed using the dlnm (19) and gam packages (20) in R software version 3.4.0.

## 3 Results

### 3.1 Descriptive statistics

During the study period, 14,259 outpatient visits for CRS were recorded. Among these cases, 7,335 CRS outpatient visits (51.44%) occurred during the cold season, while 6,924 CRS outpatient visits (48.56%) took place during the warm season. The recorded values of the daily mean temperature, mean relative humidity (RH) and daily total rainfall were 17.53°C, 76.67% and 1.28 mm, respectively. Detailed information is shown in Table 1.

**Table 1:** Summary statistics of daily outpatient visits for chronic rhinosinusitis and meteorological variables in Wuhan, China from 1 January 2018 to 31 December 2019.

Variables	N	Mean (SD)	Median	IQR	Range
Outpatient daily visits	14,259	19.67 ± 10.80	19.00	14.00	(1.00, 71.00)
Daily visits by age					
<18 years	7,160	9.87 ± 6.50	9.00	8.00	(0.00, 40.00)
18–45 years	4,360	6.01 ± 3.78	6.00	5.00	(0.00, 24.00)
45–65 years	2,174	2.99 ± 2.45	3.00	4.00	(0.00, 12.00)
>65 years	565	0.79 ± 1.05	0.00	1.00	(0.00, 6.00)
Daily visits by season					
Warm(4–10 months)	6,924	18.92 ± 9.52	18.00	13.00	(1.00,57.00)
Cold(11–3 months)	7,335	20.43 ± 11.92	19.00	15.00	(1.00,71.00)
Meteorology					
Rainfall (mm)	–	1.28 ± 4.04	0.00	0.64	(0.00, 43.50)
Temperature (°C)	–	17.53 ± 9.64	18.55	16.69	(−3.86, 33.68)
Humidity (%)	–	76.67 ± 10.14	77.12	14.09	(43.88, 99.25)

**Figure 1:**
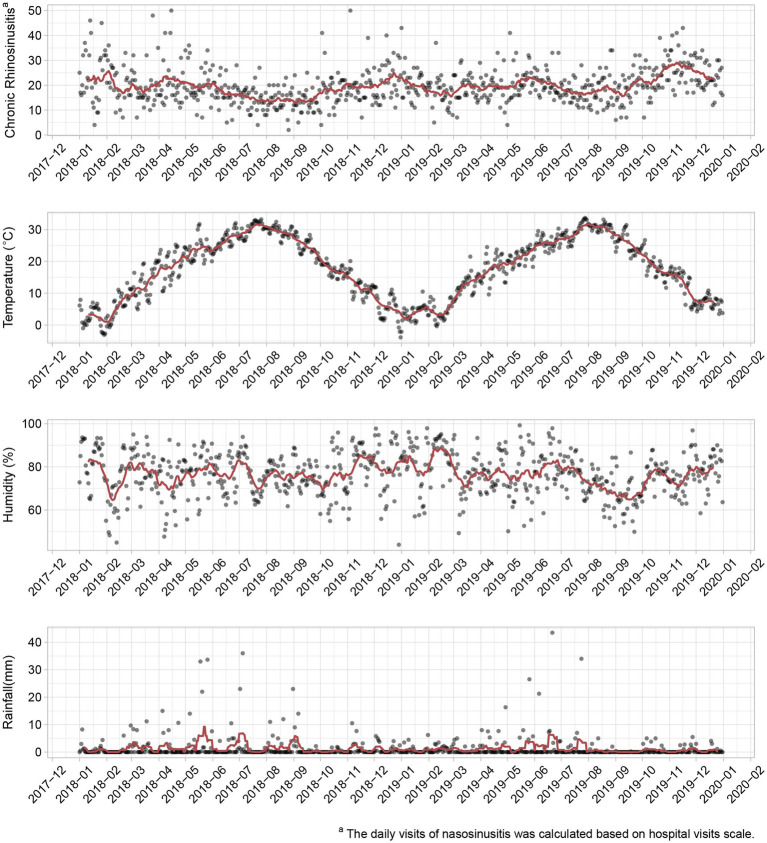
Time trends for outpatient visits of chronic rhinosinusitis (CRS), daily total rainfall (mm), mean temperature (°C), and relative humidity (%) in Wuhan, China (2018–2019). The red line represents a rolling average. Daily meteorological data were obtained from the Wuhan Meteorological Bureau.

Time trends for daily outpatient visits related to CRS and associated meteorological variations are illustrated in Figure 1. A significant seasonal trend in temperature variations is evident, with maximum temperatures consistently observed during the peak summer months of July and August, while minimum temperatures are recorded in the winter season, particularly in January and February. Notably, the recorded cases of CRS exhibit minor peaks during this same period. Furthermore, the graph indicates an increasing trend in outpatient visit volume for chronic rhinosinusitis as temperatures decreased.

Spearman’s correlations between the number of outpatient visits for CRS and various meteorological variables were presented in Supplementary Figures S1–S3. A negative correlation is observed between CRS and daily mean temperature (*r* = −0.26, *p* < 0.01), indicating a potential inverse relationship (Supplementary Figure S1). An age-stratified analysis revealed that the outpatient visit rate for CRS among children (under 18 years) and young adults (18–45 years) showed a negative correlation with the daily average temperature (Supplementary Figure S1). Specifically, an increase in outpatient visit rates was observed when temperatures decreased, suggesting that lower temperatures are associated with heightened outpatient visits in these age groups. Furthermore, no significant correlations were identified between daily mean temperature and CRS outpatient visit rates in middle-aged and older adult populations (Supplementary Figure S1). Seasonal stratification analysis indicated an inverse correlation between daily mean temperature and outpatient visits among CRS population during the warm season (Supplementary Figure S2), while no significant association was identified in the cold season (Supplementary Figure S3).

### 3.2 Results from regression models

In constructing the ultimate statistical model, we considered outpatient visits due to CRS from 2018 to 2019, totaling 14,259 cases. To capture the temporal characteristics of the outpatient visits data, the model incorporated autoregressive terms, representing the daily outpatient visits for CRS from 1 to 6 days prior. Additionally, the model allocated six degrees of freedom for the day of the year and four degrees of freedom for the number of days during the study period. This approach aimed to more accurately reflect and simulate the seasonal fluctuations and long-term trends present in the data.

**Figure 2:**
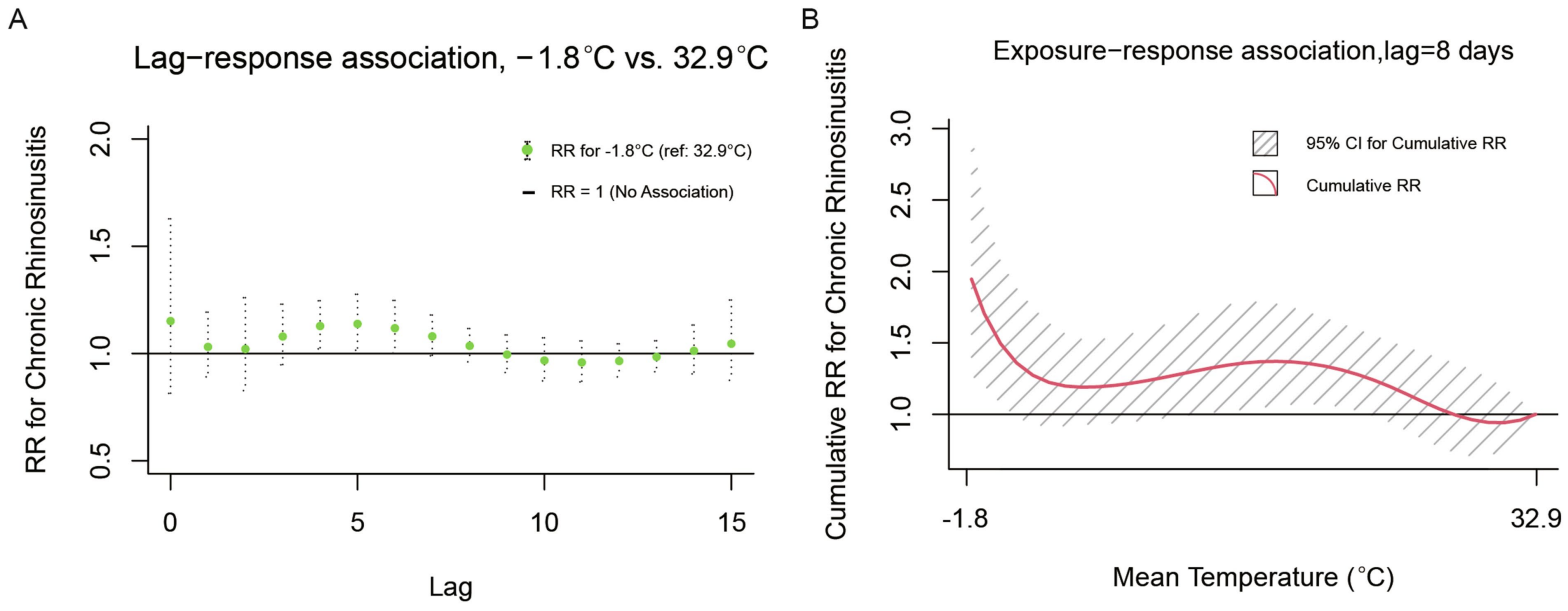
Lag-response and cumulative exposure-response relationships between daily mean temperature and outpatient visits for CRS in Wuhan. **(A)** Lag-specific relative risks associated with extreme low temperature (−1.8°C) compared to the reference temperature (32.9°C) over an 8-day lag period. **(B)** Cumulative relative risks over the 0–8 day lag period. Shaded areas represent 95% confidence intervals.

**Figure 3:**
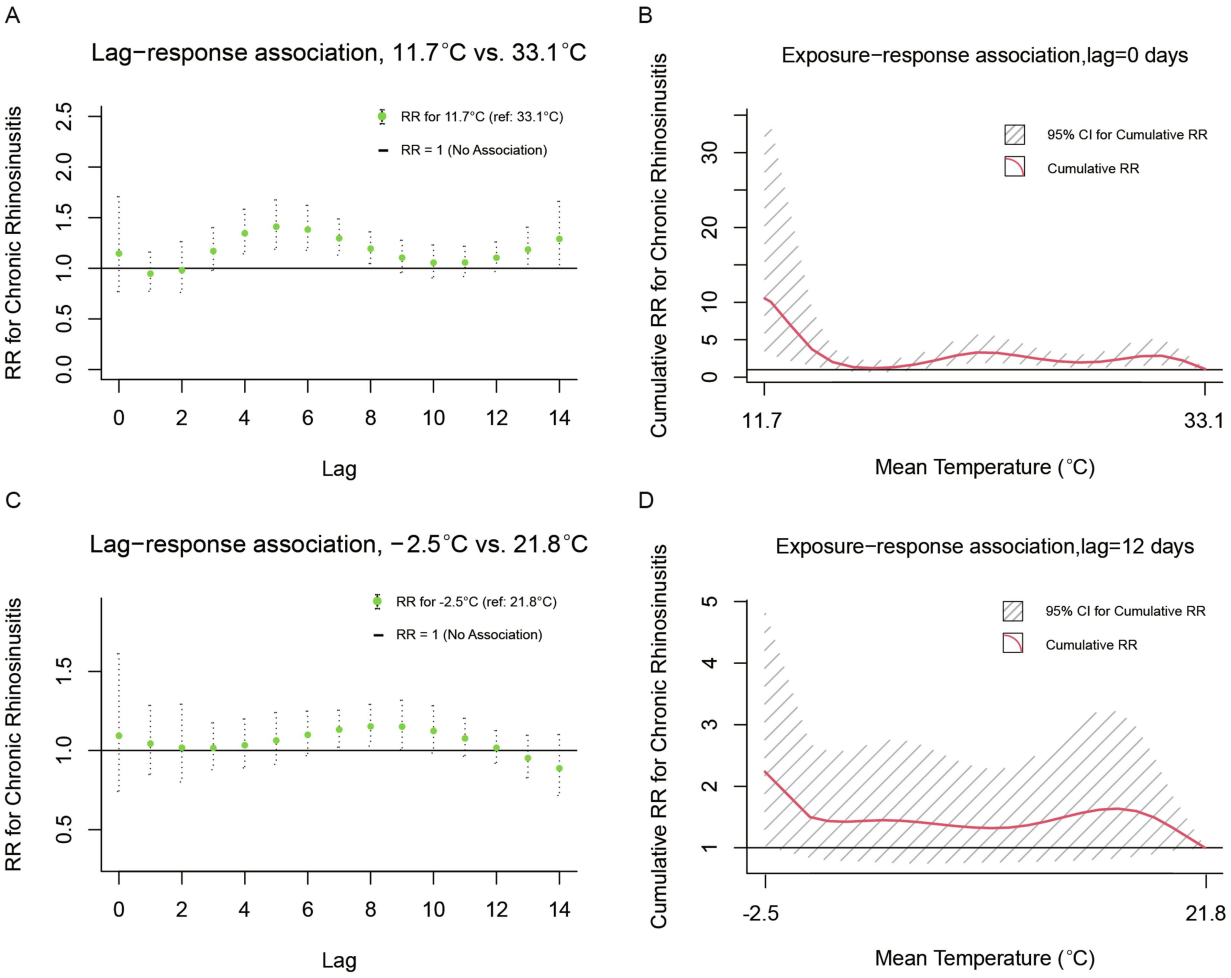
Lag-response and cumulative exposure-response associations between outpatient visits for CRS and daily mean temperature (°C) during warm and cold seasons in Wuhan. Warm season: **(A)** Lag-specific relative risks for extreme low temperature (11.7°C) compared to the reference temperature (33.1°C). **(B)** Cumulative relative risks over the entire lag period. Shaded areas represent 95% confidence intervals. Cold season: **(C)** Lag-specific relative risks for extreme low temperature (−2.5°C) compared to the reference temperature (21.8°C) over an 12-day lag period. **(D)** Cumulative relative risks over the 0–12 day lag period. Shaded areas represent 95% confidence intervals.

Our results illustrated the lag-response associations for daily mean temperature of −1.8°C (1st percentile), relative to a certain reference value of 32.9°C for CRS, and the exposure-response associations between temperature and CRS cumulative to its corresponding delayed timeframes (8 days; Figure 2). Compared to the maximum reference temperature of 32.9°C, extremely low temperature (−1.8° C) was associated with an increased risk of CRS outpatient visits from the day of exposure through the 8th day (Figure 2A). Furthermore, Figure 2B depicts the cumulative relative risks (RRs) of exposure to a specific temperature value across the entire range compared to the predefined reference value, aggregating all contributions up to 8 days, along with their 95% confidence intervals. A significant correlation was observed for temperatures ranging from −1.8°C to 28°C. Above 28°C, the risk of CRS outpatient visits decreased gradually, with extreme temperatures (−1.8°C, 1st percentile) associated with an RR of 1.946 (95% CI 1.273–2.973). Stratified analyses revealed seasonal modulation of outpatient risks for CRS (Figure 3). During warm seasons, extreme cold (11.7°C, 1st percentile) was associated with an increased risk of same-day visits, a reduced risk of visits 1–2 days post-heatwave, and a subsequent rise in risk after 3 days. The relative risk attributable to extreme cold was 10.531 (95% CI 3.216–34.482; Figures 3A,B). In the cold season, extreme cold (−2.5°C, 1st percentile) was linked to a higher risk of CRS outpatient visits for up to 12 days, compared to the reference temperature (21.8°C), and the extreme temperature was associated with a relative risk of 2.240 (95% CI 1.033–4.859; Figures 3C,D).

**Figure 4:**
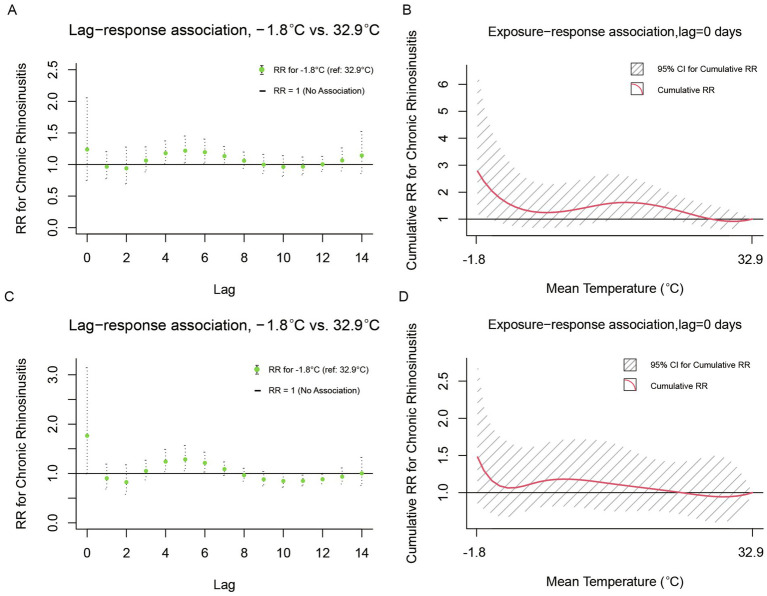
Lag-response and cumulative exposure-response associations between outpatient visits for CRS and daily mean temperature among children (under 18 years) and young adults (18–45 years) in Wuhan Children (<18 years): **(A)** Lag-specific relative risks for extreme low temperature (−1.8°C) compared to the reference temperature (32.9°C). **(B)** Cumulative relative risks over the entire lag period. Shaded areas represent 95% confidence intervals. Young adults (18–45 years): **(C)** Lag-specific relative risks for extreme low temperature (−1.8°C) compared to the reference temperature (32.9°C). **(D)** Cumulative relative risks over the entire lag period. Shaded areas represent 95% confidence intervals.

We found a distinct sensitivity to temperature variations between children (under 18 years; Figures 4A,B) and young adults (18 ~ 45 years; Figures 4C,D). The risk of CRS outpatient visits demonstrates a significant correlation with daily mean temperature, a relationship that is modulated by temporal lag and cumulative exposure. In both age groups, the lag-response curves exhibit an inverse M-shaped configuration, with the under-18 age group displaying a milder response to temperature extremes. Beginning on the day of cumulative exposure, an increase in temperature is associated with a stepwise reduction in the risk of CRS-related outpatient visits across both demographic groups.

## 4 Discussion

In this study, we investigated the associations between outpatient visits for CRS and meteorological factors such as temperature, relative humidity and precipitation in Wuhan, during the period from 2018 to 2019. Our results indicated a negative correlation between temperature and the number of outpatient visits for CRS. Furthermore, stratified analysis revealed that this negative correlation is particularly pronounced during the warm season and among children and young adults. These findings align with a previous study conducted in southwest Iran, which reported that the minimum mean annual temperature had inverse effects on CRS occurrence (21) may be attributed to the inhalation of cold air, which can cause nasal congestion and sinus dryness, thereby impairing mucociliary function and potentially leading to CRS (22). Another possible explanation for the direct association between temperature and CRS is the increased vulnerability to indoor allergens resulting from spending more time indoors, coupled with an elevated risk of viral sinus infections (22). However, some contradictory findings have also been reported. Wee et al. (11) provided robust evidence that the associations between mean, highest, and lowest temperature, as well as temperature range, and CRS show no significant correlation; however, they did not account for lag effects. Du et al. (13) also found a significant positive association between ambient temperature and chronic rhinosinusitis in United States. In addition, our study did not detect a significant association between rainfall and humidity and CRS, despite some studies suggesting that relative humidity and rainfall significantly impact CRS outpatient visits (21). Such discrepancy may be attributed to variations in geography, climate, and ethnicity.

In the stratified analysis, children (under 18 years) and young adults (18–45 years) were found to have a higher risk of CRS outpatient visits due to extreme low temperatures. The higher susceptibility in this age group may be attributed to their relatively physiological differences (23), behavioral habits (24), and climate adaptability (25). Additionally, individuals in this age group are more likely to take outdoor activities, which may consequently increase their exposure to outdoor air pollution and potentially elevate their risk of respiratory diseases. However, we did not observe a significant association between extreme temperatures and CRS outpatient visits among middle-aged individuals and the older adults. This may be related to several factors, such as the small sample size of older adults in this study, which could limit the generalizability of our findings. Additionally, the presence of multiple concurrent health issues among the older adults might obscure the impact of extreme temperatures on CRS visits. It is also possible that enhanced awareness of self-care among older adults could contribute to a reduced sensitivity to temperature variations, as they may take more proactive measures to protect their health in extreme weather conditions.

The limitations of our study also need to be acknowledged. Firstly, our study included patients from only one hospital, which may limit the generalizability of findings to populations in other regions with different social and environmental settings. Secondly, our analysis did not account for air pollution levels (e.g., PM₂.₅, NO₂) and individual behavioral factors (indoor heating usage, mask-wearing habits, and time spent outdoors), which may confound the observed temperature-CRS associations. Thirdly, as an observational study, our analysis identifies associations but cannot establish causality between meteorological factors and CRS outcomes.

## 5 Conclusion

We found that lower temperatures are associated with a significant increase in outpatient visits for CRS. Subsequent stratified analyses revealed that this inverse relationship is particularly pronounced during the warmer seasons and within specific demographic subgroups, such as children and young adults. Our findings enrich the existing epidemiological knowledge of CRS by elucidating the specific impact of temperature on outpatient visit patterns in large populations. The results underscore the necessity for healthcare providers and public health policymakers to integrate environmental risk factors—particularly extreme low temperatures into CRS management strategies, while raising public awareness of climate-health interactions. Targeted preventive interventions for high-risk groups (e.g., children and young adults) should be prioritized, including enhanced indoor thermal protection and reduced exposure to cold outdoor environments. Future multicenter studies incorporating hospitals from diverse climate zones (e.g., northern cold regions and southern coastal areas) are needed to evaluate regional variations in meteorological-CRS associations.

## Data Availability

The original contributions presented in the study are included in the article/Supplementary material, further inquiries can be directed to the corresponding authors.

## Footnotes

^1^ http://data.cma.cn

## References

[1] Kato A Schleimer RP Bleier BS. Mechanisms and pathogenesis of chronic rhinosinusitis. J Allergy Clin Immunol. (2022) 149:1491–503. doi: 10.1016/j.jaci.2022.02.016, PMID: 35245537 PMC9081253

[2] Orlandi RR Kingdom TT Smith TL Bleier B DeConde A Luong AU. International consensus statement on allergy and rhinology: rhinosinusitis 2021. Int Forum Allergy Rhinol. (2021) 11:213–739. doi: 10.1002/alr.22741, PMID: 33236525

[3] Zeng M Wang H Liao B Wang H Long XB Ma J. Clinical and biological markers predict the efficacy of glucocorticoid- and macrolide-based postoperative therapy in patients with chronic Rhinosinusitis. Am J Rhinol Allergy. (2021) 35:596–606. doi: 10.1177/1945892420982236, PMID: 33348995

[4] Hastan D Fokkens WJ Bachert C Newson RB Bislimovska J Bockelbrink A. Chronic rhinosinusitis in Europe - an underestimated disease. A GA2LEN study. Allergy. (2011) 66:1216–23. doi: 10.1111/j.1398-9995.2011.02646.x, PMID: 21605125

[5] Zhou Q Ma J Biswal S Rowan NR London NR Riley CA. Air pollution, genetic factors, and chronic rhinosinusitis: a prospective study in the UK biobank. Sci Total Environ. (2024) 940:173526. doi: 10.1016/j.scitotenv.2024.173526, PMID: 38825199

[6] Leland EM Vohra V Seal SM Zhang Z Ramanathan M Jr. Environmental air pollution and chronic rhinosinusitis: a systematic review. Laryngoscope Investig Otolaryngol. (2022) 7:349–60. doi: 10.1002/lio2.774, PMID: 35434330 PMC9008184

[7] Burte E Leynaert B Marcon A Bousquet J Benmerad M Bono R. Long-term air pollution exposure is associated with increased severity of rhinitis in 2 European cohorts. J Allergy Clin Immunol. (2020) 145:834–842.e6. doi: 10.1016/j.jaci.2019.11.040, PMID: 31983528

[8] Park M Lee JS Park MK. The effects of air pollutants on the prevalence of common ear, nose, and throat diseases in South Korea: a National Population-Based Study. Clin Exp Otorhinolaryngol. (2019) 12:294–300. doi: 10.21053/ceo.2018.00612, PMID: 30813711 PMC6635704

[9] Lu M Ding S Wang J Liu Y An Z Li J. Acute effect of ambient air pollution on hospital outpatient cases of chronic sinusitis in Xinxiang, China. Ecotoxicol Environ Saf. (2020) 202:110923. doi: 10.1016/j.ecoenv.2020.110923, PMID: 32800210

[10] Kim J Waugh DW Zaitchik BF Luong A Bergmark R Lam K. Climate change, the environment, and rhinologic disease. Int Forum Allergy Rhinol. (2023) 13:865–76. doi: 10.1002/alr.23128, PMID: 36575965

[11] Wee JH Min C Jung HJ Park MW Park B Choi HG. Association between air pollution and chronic rhinosinusitis: a nested case-control study using meteorological data and national health screening cohort data. Rhinology. (2021) 59:451–9. doi: 10.4193/Rhin21.141, PMID: 34472546

[12] AlQahtani A Alim B Almudhaibery F Mulafikh D Almutairi S Almohanna S. The impact of climatic, socioeconomic, and geographic factors on the prevalence of allergic fungal Rhinosinusitis: a worldwide ecological study. Am J Rhinol Allergy. (2022) 36:423–31. doi: 10.1177/19458924211069226, PMID: 35187957

[13] Du R Jiao W Ma J Zhou Q Liang ZS Sun S. Association between ambient temperature and chronic rhinosinusitis. Int Forum Allergy Rhinol. (2023) 13:1906–14. doi: 10.1002/alr.23152, PMID: 36897288

[14] Wang YC Lin YK. Temperature effects on outpatient visits of respiratory diseases, asthma, and chronic airway obstruction in Taiwan. Int J Biometeorol. (2015) 59:815–25. doi: 10.1007/s00484-014-0899-0, PMID: 25225115

[15] okkens WJLV Hopkins C. European position paper on rhino sinusitis and nasal polyps 2020. Rhinology. (2020) 58:464. doi: 10.4193/Rhin20.600 32077450

[16] Lan J Huang Q Yang L Li Y Yang J Jiang B. Effects of ambient air pollution on outpatient visits for psoriasis in Wuhan, China: a time-series analysis. Br J Dermatol. (2023) 188:491–8. doi: 10.1093/bjd/ljac124, PMID: 36641781

[17] GBD 2013 Mortality and Causes of Death Collaborators. Mortality GBD, causes of death C. Global, regional, and national age-sex specific all-cause and cause-specific mortality for 240 causes of death, 1990-2013: a systematic analysis for the global burden of disease study 2013. Lancet. (2015) 385:117–71. doi: 10.1016/S0140-6736(14)61682-2 25530442 PMC4340604

[18] Wang P Goggins WB Chan EYY. A time-series study of the association of rainfall, relative humidity and ambient temperature with hospitalizations for rotavirus and norovirus infection among children in Hong Kong. Sci Total Environ. (2018) 643:414–22. doi: 10.1016/j.scitotenv.2018.06.189, PMID: 29940452

[19] Gasparrini A. Distributed lag linear and non-linear models in R: the package dlnm. J Stat Softw. (2011) 43:1–20. doi: 10.18637/jss.v043.i08 PMC3191524 22003319

[20] Pedersen EJ Miller DL Simpson GL Ross N. Hierarchical generalized additive models in ecology: an introduction with mgcv. PeerJ. (2019) 7:e6876. doi: 10.7717/peerj.6876, PMID: 31179172 PMC6542350

[21] Ghatee MA Kanannejad Z Nikaein K Fallah N Sabz G. Geo-climatic risk factors for chronic rhinosinusitis in Southwest Iran. PLoS One. (2023) 18:e0288101. doi: 10.1371/journal.pone.0288101, PMID: 37406025 PMC10321645

[22] Jaume F Valls-Mateus M Mullol J. Common cold and acute Rhinosinusitis: up-to-date management in 2020. Curr Allergy Asthma Rep. (2020) 20:28. doi: 10.1007/s11882-020-00917-5, PMID: 32495003 PMC7266914

[23] Rotulo GA Palma P. Understanding COVID-19 in children: immune determinants and post-infection conditions. Pediatr Res. (2023) 94:434–42. doi: 10.1038/s41390-023-02549-7, PMID: 36879079 PMC9987407

[24] Trakoli A. Raising awareness of the health effects of environmental exposures. BMJ. (2019) 365:l1878. doi: 10.1136/bmj.l1878, PMID: 31028019

[25] Campbell-Lendrum D Neville T Schweizer C Neira M. Climate change and health: three grand challenges. Nat Med. (2023) 29:1631–8. doi: 10.1038/s41591-023-02438-w, PMID: 37464036

