# Supplementary material for: Effects of meteorological factors on outpatient visits for chronic rhinosinusitis in Wuhan, China (2018–2019): a time-series analysis
Source: Front Public Health. 2025 Jun 27;13:1621856. doi: 10.3389/fpubh.2025.1621856 (PMC12245921; doi:10.3389/fpubh.2025.1621856)

Figure S1 Spearman’s correlations between the number of outpatient visits for CRS and various meteorological variables


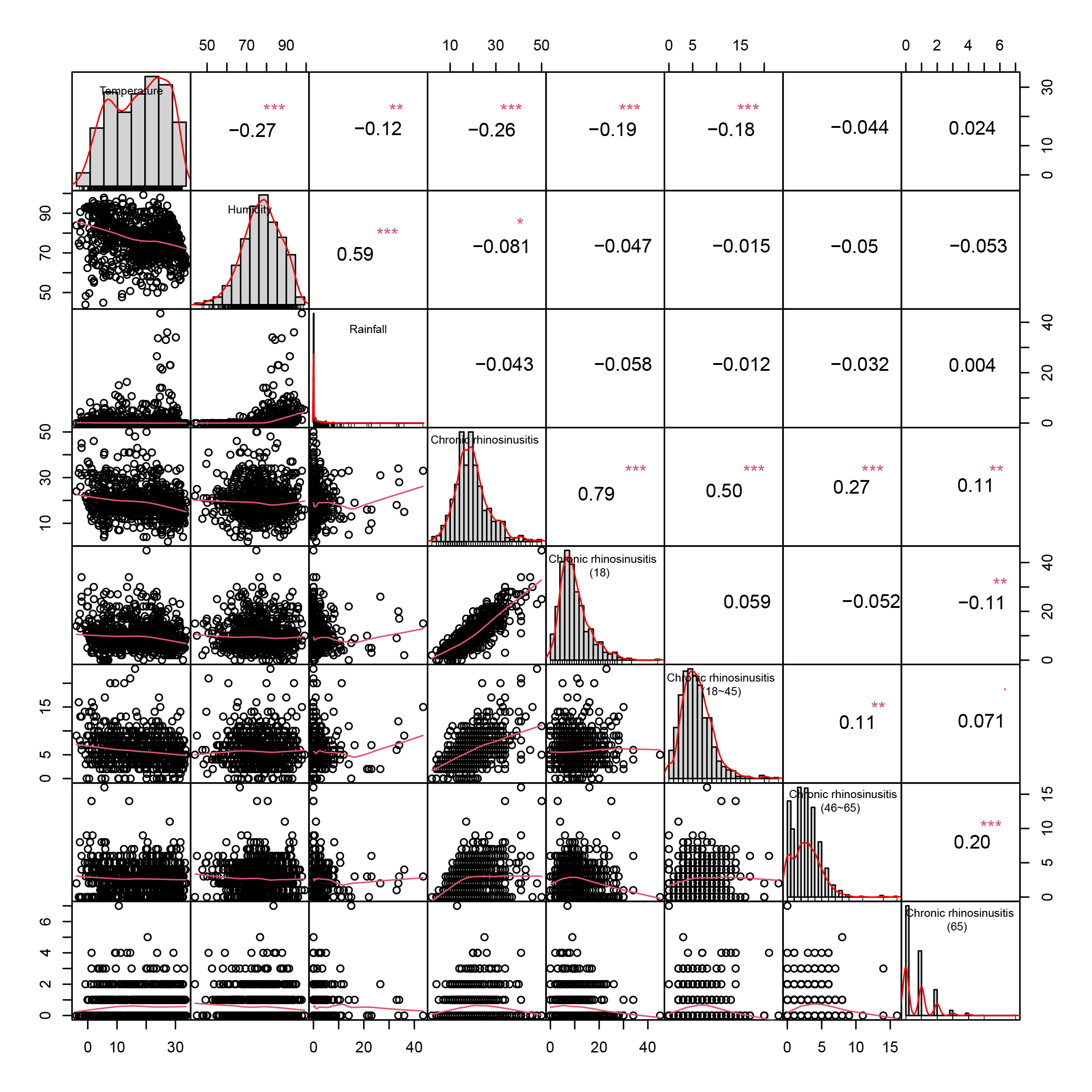


Figure S2 Spearman’s correlations between the number of outpatient visits for CRS and temperature in warm season


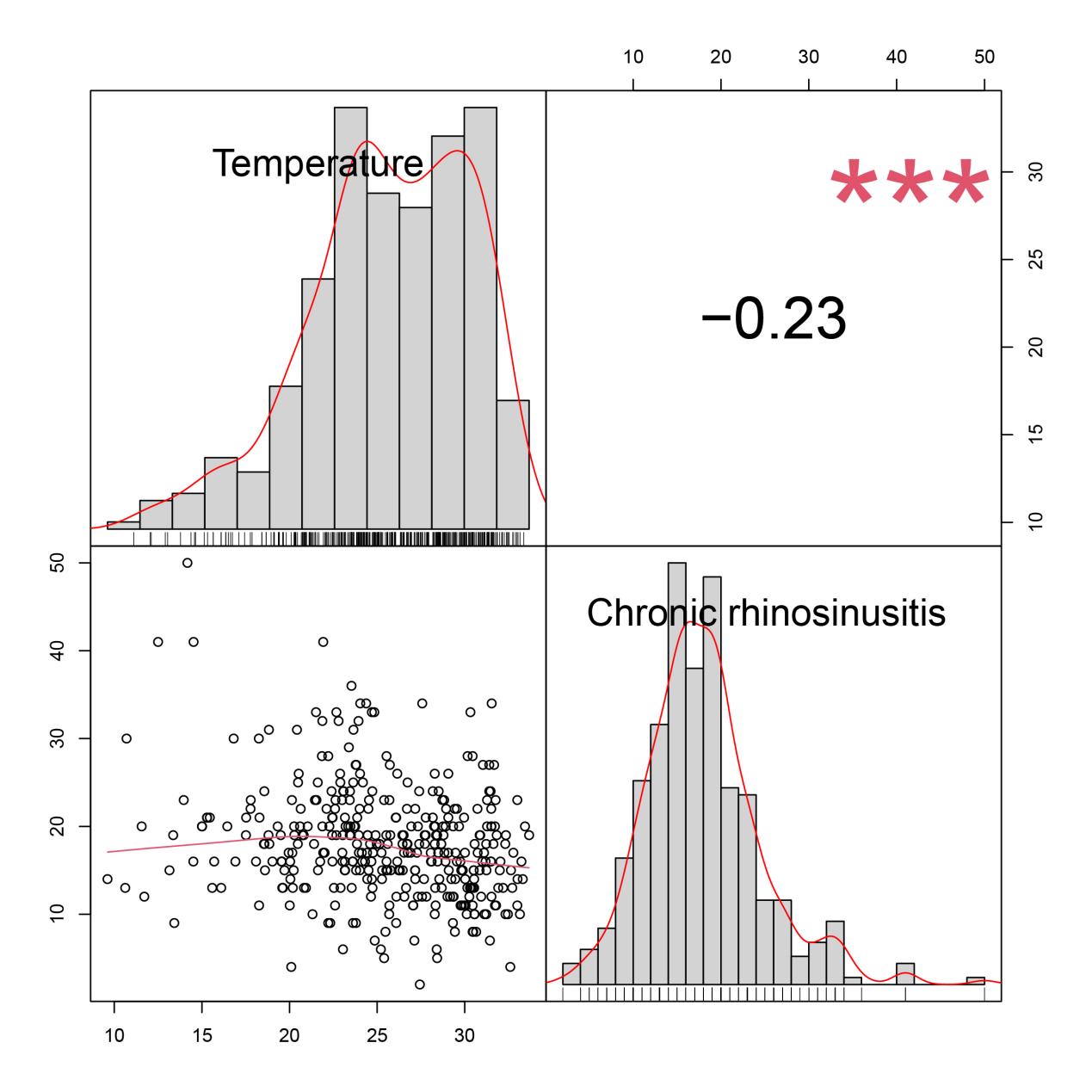


Figure S3 Spearman’s correlations between the number of outpatient visits for CRS and temperature in cold season


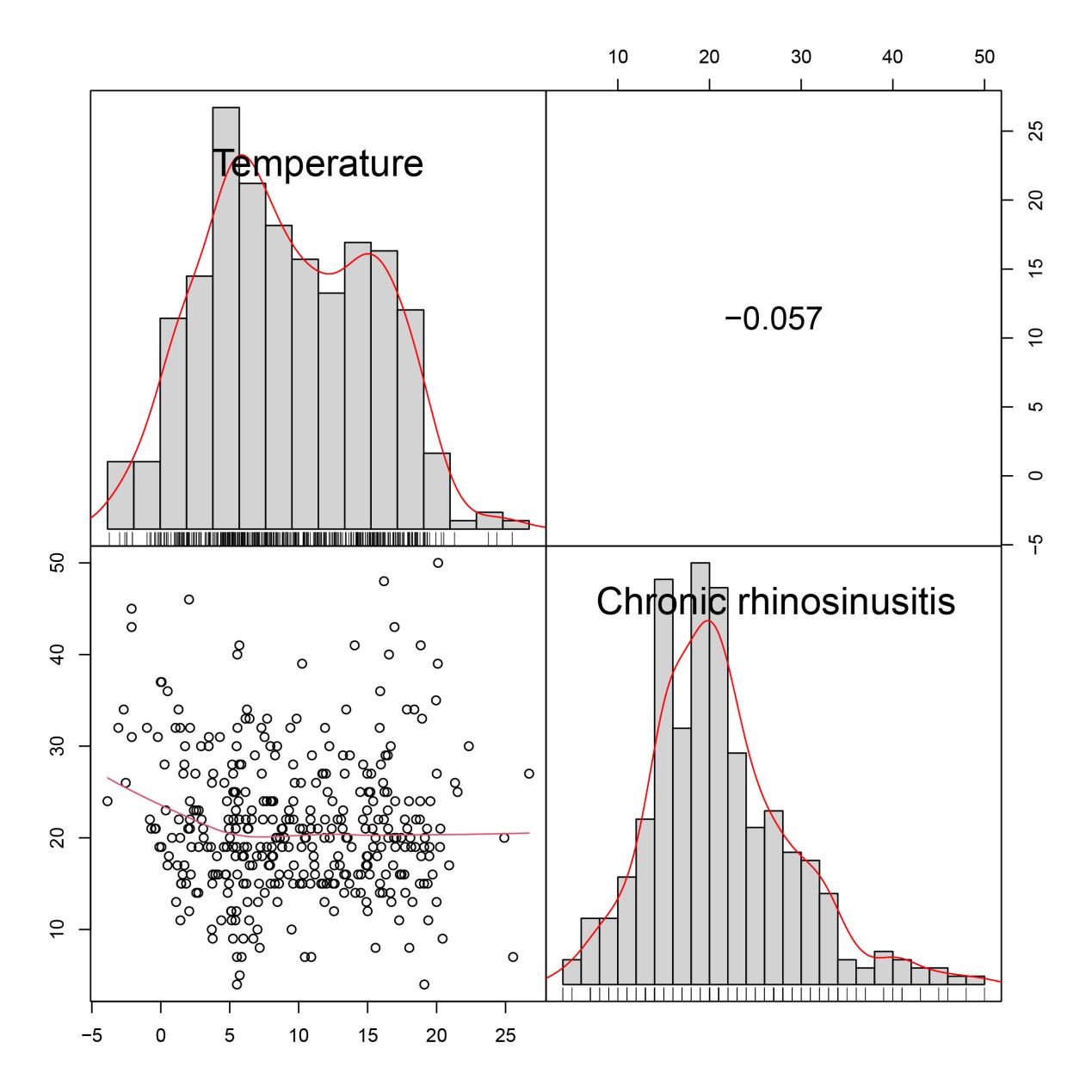

Supplement: Supplementary file 1 [file Data_Sheet_1.docx]
